# The hidden cost of hypotension: redefining hemodynamic management to improve patient outcomes

**DOI:** 10.1016/j.bjane.2024.844581

**Published:** 2024-12-05

**Authors:** Eric B. Lineburger, Deepak K. Tempe, Luiz Guilherme V. da Costa, G. Burkhard Mackensen, Fabio V. Papa, Carlos Galhardo, Mohamed R. El Tahan, Marcello F. Salgado-Filho, Rodrigo Diaz, André P. Schmidt

**Affiliations:** a Hospital São José, Departamento de Anestesia e Tratamento da Dor, Criciúma, SC, Brazil; b Hospital São José, Centro de Pesquisa, Criciúma, SC, Brazil; c Universidade do Extremo Sul Catarinense, Criciúma, SC, Brazil; d Institute of Liver and Biliary Sciences, New Delhi, India; e Hospital Israelita Albert Einstein, Departamento de Anestesiologia, São Paulo, SP, Brazil; f UW Medicine, Department of Anesthesiology & Pain Medicine, Washington, USA; g University of Toronto, St. Michael's Hospital, Department of Anaesthesia, Toronto, Canada; h McMaster University, Hamilton Health Sciences, Department of Anesthesia, Hamilton, Canada; i Mansoura University, College of Medicine, Department of Anaesthesia and Surgical Intensive Care, Mansoura, Egypt; j Imam Abdulrahman Bin Faisal University, College of Medicine, Cardiothoracic Anaesthesia, Anesthesiology Department, Dammam, Saudi Arabia; k Universidade Federal do Rio de Janeiro (UFRJ), Rio de Janeiro, RJ, Brazil; l Universidade Federal do Rio Grande do Sul (UFRGS), Hospital de Clínicas de Porto Alegre (HCPA), Serviço de Anestesia e Medicina Perioperatória, Porto Alegre, RS, Brazil; m Santa Casa de Porto Alegre, Serviço de Anestesia, Porto Alegre, RS, Brazil; n Hospital Nossa Senhora da Conceição, Serviço de Anestesia, Porto Alegre, RS, Brazil; o Programa de Pós-graduação em Ciências Pneumológicas e Programa de Pós-graduação em Ciências Cirúrgicas, UFRGS; p Faculdade de Medicina da Universidade de São Paulo (FMUSP), Programa de Pós-Graduação em Anestesiologia, Ciências Cirúrgicas e Medicina Perioperatória, São Paulo, SP, Brazil

Traditionally, the approach to hemodynamic stability during anesthesia has focused on maintaining an appropriate depth of anesthesia and avoiding extreme fluctuations in blood pressure (BP). Significant but brief BP changes are often managed with rapid administration of vasopressors or vasodilators, without fully considering their impact on patient safety and outcomes. However, growing evidence suggests that even short episodes of intraoperative hypotension, lasting only a few minutes, can negatively affect clinical outcomes.^1^, ^2^, ^3^, ^4^, ^5^

Is this surprising? Suppose a healthy individual suddenly experiences a systolic BP of around 70 mmHg. In that case, he/she will likely seek a place to sit down and wait for symptoms such as dizziness, nausea, or vertigo to subside. The older the person, the more pronounced these symptoms are likely to be. Under anesthesia, patients are unable to express discomfort or alert the surgical team about the feelings of hypotension. Therefore, anesthesiologists should maintain rigorous, continuous BP monitoring and proactively prevent and manage hypotension to ensure patient safety.

Recent European Society of Cardiology guidelines^6^ for managing elevated BP and hypertension recognize and address the adverse effects of perioperative hypotension and recommend avoiding large perioperative fluctuations in BP. They also suggest using preoperative ambulatory BP as a baseline. However, implementing these recommendations is challenging.

Perioperative hypotension is influenced by factors such as patient health, anesthesia, and surgery type, with higher risks linked to advanced age, low preoperative BP, and use of antihypertensives. Causes of intraoperative hypotension include depth of anesthesia, vasodilation, and blood loss, while postoperative hypotension may occur due to ischemia, hypovolemia, infections, or other complications.^7^

The primary adverse outcomes associated with intraoperative hypotension are acute kidney injury (AKI), myocardial injury after noncardiac surgery (MINS), delirium, stroke, hospital readmissions, and increased mortality in both cardiac and noncardiac procedures, among others.^8^, ^9^, ^10^, ^11^, ^12^, ^13^, ^14^, ^15^, ^16^ These conditions often lead to further complications, such as the progression to chronic kidney disease in cases of AKI, which can affect life expectancy.^17^

To date, there is no clear definition of perioperative hypotension. Still, most studies define it as a decrease of more than 10% to 20% from baseline values, a systolic pressure < 90 mmHg, or a MAP < 60 mmHg.^18^^,^^19^ Additionally, the number and duration of hypotensive episodes should be considered. Salmasi et al.^16^ found that more than 13 cumulative minutes with MAP < 65 mmHg increased the risk of MINS and AKI, with half of the study patients experiencing episodic drops below this threshold.

In a large retrospective cohort study of 166,091 operations, Schnetz et al.^20^ developed a risk model to evaluate intraoperative hypotension by analyzing over 7.3 million intraoperative MAP measurements. They observed that the risk of intraoperative hypotension, defined as MAP < 65 mmHg, increased exponentially as MAP approached this threshold. For instance, MAP values of 70 mmHg were associated with a fourfold higher risk of intraoperative hypotension episodes than 80 mmHg despite both values being within reference ranges. These findings highlight the need for increased vigilance, especially in patients with elevated preoperative risk factors, even when MAP levels are within traditional reference ranges.

Wesselink et al.^21^ conducted a systematic review of 42 studies analyzing different MAP thresholds and their impact on outcomes to assess the association between intraoperative hypotension and adverse postoperative outcomes in noncardiac operations. They found that brief exposures to MAP < 70 mmHg slightly increased the risk of organ injury, while prolonged exposures (10 minutes or more) to MAP < 80 mmHg further increased this risk. When MAP remained between 60 and 65 mmHg for 5 minutes or more, the risk of AKI and MINS became significant, and any exposure to MAP < 50 and 55 mmHg was associated with a higher risk of stroke and delirium.

Marcucci et al.^22^ conducted a randomized study (PeriOperative ISquemic Evaluation (POISE)-3) involving 7,490 patients on long-term antihypertensive therapy undergoing noncardiac surgery. They compared strategies aimed at avoiding hypotension (target MAP ≥ 80 mmHg) vs hypertension (target MAP ≥ 60 mmHg). Both groups showed similar rates of vascular complications, indicating that maintaining a higher MAP target did not reduce complications. However, the hypotension-avoidance group had a lower incidence of clinically significant intraoperative hypotension episodes. Although the groups had significantly different cumulative durations of hypotension (< 60 mmHg), a post-hoc analysis confirmed that the differences in vascular outcomes between the groups were not statistically significant.^23^

Several studies have reported a high incidence of intraoperative hypotension episodes, with rates ranging from around 20% to 30% in cohorts of thousands of patients primarily undergoing noncardiac operations.^3^^,^^24^, ^25^, ^26^ Thus, it is clear that maintaining BP levels above intraoperative hypotension thresholds is challenging. Although the oscillometric method is considered the standard for intraoperative BP measurement, it has significant limitations, such as the possibility of “blind spots” between measurements. One potential solution is continuous, noninvasive BP monitoring using pulse wave analysis with finger-cuff technologies.^27^ Although studies show mixed results regarding the correlation between finger-cuff measurements and invasive BP readings,^28^, ^29^, ^30^, ^31^, ^32^ finger-cuff technologies could eliminate “blind spots” in MAP measurements and provide relevant hemodynamic information to support goal-directed hemodynamic therapy (GDHT). By providing metrics such as stroke volume, stroke volume variation, cardiac output, and vascular resistance, they could help identify the underlying causes of intraoperative hypotension and guide targeted interventions.^33^^,^^34^ Unfortunately, widespread adoption of this technology is limited by high costs and scientific validation challenges.

Although the current literature compares GDHT with several postoperative outcomes, evidence linking it directly to a reduction in intraoperative hypotension episodes is sparse.^33^^,^^35^^,^^36^ Alternatively, emerging monitoring modalities based on artificial intelligence (AI) and machine learning show potential when integrated with GDHT.^37^ One notable example is the Hypotension Prediction Index (HPI), which calculates the probability of an imminent hypotensive event on a scale from 0 to 100. The HPI identifies patterns suggesting a decrease in BP and indicates the underlying causes, allowing precise, targeted interventions. It can be used with both invasive and noninvasive BP monitoring, although its algorithm remains a topic of debate.^30^^,^^38^, ^39^, ^40^

In general, trials involving HPI have shown an effective reduction in the duration of intraoperative hypotension, but they are not yet sufficiently powered to assess complex outcomes, such as major morbidity and mortality.^41^ A small observational study^42^ demonstrated that the areas under the receiver operating characteristic curve for the HPI (0.89) and concurrent MAP (0.88) were nearly identical in predicting hypotension within 5 minutes. This finding suggests that adjusting MAP alarm thresholds to 72 or 73 mmHg could serve as an alternative to using the HPI. However, research on these technologies faces significant challenges, including a lack of standardized interventions and outcomes. Variations in protocol adherence, especially in multicenter studies, complicate the ability to draw unbiased and consistent conclusions.

Recent evidence supports the use of cerebral monitoring during anesthesia to guide hemodynamic optimization.^43^^,^^44^ Since the seminal studies on the "triple low" condition, characterized by the concurrent presence of a low bispectral index (< 45), low MAP (< 75 mmHg), and low minimum alveolar concentration (MAC) (< 0.8), and its association with adverse outcomes such as increased mortality, there has been growing recognition of the importance of effectively managing both the depth of anesthesia and hemodynamic disturbances to normalize altered brain wave patterns.^45^^,^^46^ In many patients, the lower limit of the cerebral blood flow autoregulation curve shifts to the right, a phenomenon that can be identified through cerebral monitoring during anesthesia.^47^ This shift is especially relevant when burst suppression (BS) on processed electroencephalography (pEEG) or desaturation on cerebral oximetry is detected, as these abnormalities frequently resolve with MAP increases in both cardiac and noncardiac operations.^48^^,^^49^

For instance, a study by Georgii et al.^50^ demonstrated that pEEG-guided interventions during anesthesia, which involve adjusting MAP and anesthetic concentrations, significantly reduced the duration and intensity of BS in older patients. In 55% of cases, correcting MAP alone was sufficient to resolve BS episodes, suggesting that hypotension may be an underlying cause. Similarly, Thomsen et al.^51^ conducted a study in which patients undergoing vascular surgery were randomized to receive either pEEG-guided general anesthesia or standard anesthesia. Norepinephrine was administered to maintain MAP > 65 mmHg to prevent intraoperative hypotension. The results showed that the pEEG-guided group required about one-third less norepinephrine, likely due to lighter anesthesia reducing the hypotension typically induced by anesthetic agents. Although still showing inconsistent outcome results, intraoperative brain monitoring can help guide hypotension treatment.^52^, ^53^, ^54^ Payne et al.^52^ conducted a systematic review and meta-analysis of randomized controlled trials and found that depth-of-anesthesia monitoring may reduce postoperative mortality, although further research is needed to confirm these findings.

Attention should also be given to the entire perioperative period, extending vigilant BP management to the postoperative period. Smischney et al.,^55^ in a multicenter retrospective study, analyzed data from 3,185 noncardiac surgery patients who did not experience intraoperative hypotension (defined as MAP ≤ 65 mmHg) and were discharged from the intensive care unit after 48 hours. They found that postoperative hypotension (MAP ≤ 65 mmHg and ≤ 55 mmHg) was associated with an increased risk of critical adverse events within 30 and 90 days. MAP ≤ 65 mmHg raised the likelihood of severe cardiac or cerebrovascular events, while MAP ≤ 55 mmHg further increased this risk and was associated with higher mortality. Additionally, MAP ≤ 55 mmHg was also linked to a higher risk of advanced-stage AKI. These findings are consistent with those by Marcucci et al.,^22^ who did not find differences in vascular outcomes when treating intraoperative hypotension. The authors emphasized the importance of further research into postoperative hypotension as a contributor to organ injury.

Therefore, it is evident that maintaining BP stability requires a collaborative perioperative effort, with continuous vigilance. In the real world, is such engagement feasible in current clinical practice? Until the current paradigm shifts, certain efforts may prove valuable. In a study involving postcardiac surgery patients, Desebbe et al.^56^ showed that those managed with a closed-loop vasopressor (CLV) system spent only 1.4% of the time in hypotension (MAP < 65 mmHg) compared to 12.5% with manual control. The CLV system maintained MAP within the target range (65-75 mmHg) 95% of the time vs 66% with manual control, with significantly more infusion adjustments, demonstrating its effectiveness. Hence, the use of AI-based predictive algorithms such as the HPI and advanced monitoring techniques could revolutionize perioperative care. Nevertheless, the validation and clinical implementation of these technologies remain challenging.

A novel concept termed “protective hemodynamics” has emerged, focused on minimizing the risks associated with hypotension interventions.^57^ The objective of this approach is to reduce harm from excessive vasoconstriction while prioritizing patient outcomes rather than rigidly adhering to BP targets. It involves the implementation of dynamic BP targets that decrease in response to increasing vasopressor dosages. The BP target should be maintained within the reference range, particularly when vasopressor use is minimal or absent. As vasopressor dosage increases, lower BP targets should be accepted. This adaptive strategy aims to reduce iatrogenic harm linked to high doses of vasopressors by minimizing the risk of adverse effects. Elevated mean BP values due to increased doses of catecholamines are associated with increased mortality rates.^58^ A recent meta-analysis comparing permissive vs targeted intraoperative MAP targets showed that MAP ≥ 60 mm Hg was not always equal to end-organ perfusion, whereas MAP ≤ 60 mm Hg was not associated with increased mortality or adverse effects.^59^ These findings promoted the development of the C.L.E.A.R. protective hemodynamics approach for organ protection: a) **C**ustomize targets (e.g., reduce and individualize MAP targets and avoid overtreatment), b) **L**imit catecholamines (e.g., minimize the use of catecholamines, consider drugs acting on different receptors, and administer the right vasopressor to the right patient at the right time), c) **E**nhance flow focusing on microcirculation and regional blood flow, d) **A**djust fluid balance guided by echocardiography with optimal cardiac output and microvascular blood flow, and e) **R**esolve underlying conditions (e.g., with the use of steroids, sedation, and temperature control).^57^ The impact of this approach needs to be validated in multicenter randomized control trials.

In summary, intraoperative hypotension is a frequent challenge during surgery and is linked to significant adverse outcomes, including an increased risk of mortality. Effective perioperative BP management is essential in modern anesthesia practice. A key area requiring further investigation is the routine application of a uniform low MAP threshold of 65 mmHg for most patients, without accounting for individualized targets tailored to pressure, flow, and oxygen delivery needs. The integration of algorithm-driven hemodynamic monitoring systems, combined with a redefined approach to hemodynamic stability, has the potential to support intraoperative and postoperative teams in optimizing patient outcomes.

## Conflicts of interest

The authors declare no conflicts of interest.
